# Neutrophil-to-Lymphocyte Ratio Is Not Associated with Severity of Coronary Artery Disease and Is Not Correlated with Vitamin D Level in Patients with a History of an Acute Coronary Syndrome

**DOI:** 10.3390/biology11071001

**Published:** 2022-07-01

**Authors:** Ewelina A. Dziedzic, Jakub S. Gąsior, Agnieszka Tuzimek, Marek Dąbrowski, Piotr Jankowski

**Affiliations:** 1 Medical Faculty, Lazarski University in Warsaw, 02-662 Warsaw, Poland; 2 Centre of Postgraduate Medical Education, Department of Internal Medicine and Geriatric Cardiology, 01-813 Warsaw, Poland; agnieszkatuzimek@gmail.com (A.T.); piotrjankowski@interia.pl (P.J.); 3 Department of Pediatric Cardiology and General Pediatrics, Medical University of Warsaw, 02-091 Warsaw, Poland; jgasior@wum.edu.pl; 4 Department of Cardiology, Bielanski Hospital, 01-809 Warsaw, Poland; mardab@vp.pl; 5 Center of Postgraduate Medical Education, Department of Epidemiology and Health Promotion, School of Public Health, 01-826 Warszawa, Poland

**Keywords:** neutrophil-to-lymphocyte ratio, coronary artery disease, myocardial infarction, Coronary Artery Surgery Study Score, vitamin D

## Abstract

**Simple Summary:**

Coronary artery disease (CAD), the leading cause of death worldwide, is caused by atherosclerosis. Atherosclerosis has an inflammatory component, which can be measured with the neutrophil-to-lymphocyte ratio (NLR). Vitamin D has anti-inflammatory and anti-atherogenic properties that affect many mechanisms involved in CAD. In this study, we investigated the association between NLR, vitamin D levels, and the severity of CAD in a group of patients who had a myocardial infarction (MI) in the past. Our results show that patients with acute coronary syndrome had a higher NLR compared to those with stable CAD. No associations were observed between NLR and the severity of CAD. We found no correlation between vitamin D levels and NLR. NLR could be used as a prognostic marker of consecutive MI in patients with CAD and previous MI.

**Abstract:**

Coronary artery disease (CAD), the leading cause of death worldwide, has an underlying cause in atherosclerosis. The activity of this inflammatory process can be measured with neutrophil-to-lymphocyte ratio (NLR). The anti-inflammatory and anti-atherogenic properties of vitamin D affect many mechanisms involved in CAD. In this study, we investigated the association between NLR, vitamin D concentration, and severity of CAD in a group of patients with a history of myocardial infarction (MI). NLR was higher in patients with acute coronary syndrome (ACS) in comparison to those with stable CAD (median: 2.8, range: 0.96–24.3 vs. median: 2.3, range: 0.03–31.6; *p* < 0.05). No associations between NLR and severity of CAD (*p* = 0.14) in the cohort and in the subgroups with stable CAD (*p* = 0.40) and ACS (*p* = 0.34) were observed. We found no correlation between vitamin D level and NLR neither in the whole study group (*p* = 0.29) nor in subgroups of patients with stable CAD (*p* = 0.84) and ACS (*p* = 0.30). NLR could be used as prognostic biomarker of consecutive MI in patients with CAD and a history of MI.

## 1. Introduction

Coronary Artery Disease (CAD) is the leading cause of death worldwide. As stated by the World Health Organization (WHO), CAD was responsible for 16% of all deaths in the world in 2019, which results in it being the second greatest cause of disability-adjusted life years [1]. Atherosclerosis, which plays an important role in the onset and progression of CAD and its complications, was found to have a significant inflammatory component [2]. An initial injury of the endothelium activates the atherogenic process not only by macrophage activation and foam cell formation [3] but also by releasing a cascade of inflammatory mechanisms, including activation of inflammatory cells [4], which contribute to the further release of cytokines [5] and the overproduction of metalloproteinases destabilizing an atherosclerotic plaque [6]. Evaluation of inflammatory markers is considered to be valuable in predicting the outcome of patients with CAD, as it was established that elevated levels of various inflammatory markers are associated with plaque instability [7], cardiovascular risk [8], mortality [9] as well as the extent of CAD and myocardial perfusion [10,11].

White blood cell count, a widely available inflammatory marker, was previously associated with a greater extent of CAD and increased mortality in patients with unstable angina or non-ST segment elevation myocardial infarction (MI) [12]. As lymphocytes infiltrate the atherosclerotic plaque and release a variety of pro- and anti-inflammatory cytokines [13], neutrophils, the first line of defense under acute inflammatory response, promote macrophage activation and plaque destabilization [14]. Its increase in count was also associated with the severity of CAD [15]. The neutrophil-to-lymphocyte ratio (NLR) provides an insight into the imbalance of immune pathways of inflammation, measured by neutrophil count, and the stress response, measured by lymphocyte count [16,17]. As a representative inflammatory marker, NLR was described as correlating with more frequent cardiac events [18], as well as with the angiographic severity of CAD [19], mortality [20], and restenosis in the stent [21].

Vitamin D has diverse properties, among which its anti-inflammatory and anti-atherogenic effects are of particular interest [22]. It modifies the immune response by shifting away from pro-atherogenic T helper (Th) cells 1 and toward anti-atherogenic Th2 cells as well as production of their anti-inflammatory cytokines, including other immunomodulatory mechanisms [23]. Although the effect of vitamin D on neutrophils is not as clearly established as on lymphocytes, it was shown that vitamin D reduces the production of pro-inflammatory mediators and the formation of reactive oxygen species in neutrophils while boosting anti-pathogenic activity [24]. Vitamin D also affects a wide variety of cells of the vessel wall (including endothelial and vascular smooth muscle cells) as well as other mechanisms involved in atherogenesis, such as insulin resistance, lipid profile, and the renin-angiotensin-aldosterone system [23].

Although the current indications to supplement vitamin D in patients with CAD are not different than in the general population [25], there are some reports of influence of vitamin D supplementation on CAD severity [26,27] and risk of mortality or other cardiovascular events [28,29]. The results of the available studies indicate a relationship between low serum vitamin D concentration and cardiovascular diseases [30,31,32], suggesting an influence on the established risk factors for coronary disease [33], vascular endothelial dysfunction [32], the severity of coronary atherosclerosis [30,32] as well as overall and cardiovascular mortality [34]. However, the available data on the relationship between markers of inflammation, which underlies atherosclerosis and cardiovascular disease, and vitamin D levels in patients with CAD are very limited [32,33,35,36]. The authors suggest a relationship between low serum vitamin D concentration and elevated inflammatory markers such as WBC, NLR [35] or CRP [35,36], indicating an improvement in endothelial function [32] and a decrease in inflammatory markers as a result of calcitriol supplementation [33]. Thus, the main focus of this study was to investigate the association between inflammation activity as measured by NLR, vitamin D concentration, and severity of CAD in a group of patients with a history of MI.

## 2. Materials and Methods

### 2.1. Population

The study is based on data from 268 patients who were hospitalized in the Cardiology Department of Bielanski Hospital, Warsaw, Poland, and underwent diagnostic catheter angiography to evaluate the extent of CAD between 2013 and 2017. The final statistical analysis included 181 men and 87 women, whose ages ranged from 36 to 93 years, with a history of a previous myocardial infarction treated with acetylsalicylic acid and atorvastatin or rosuvastatin. Details on clinical characteristics of the patients were presented in our previous articles [37,38,39,40,41,42], as this study is a part of an ongoing project addressing the vitamin D association with the severity of CAD in Polish patients. Exclusion criteria included platelet count <100 or >450 × 10^3^ µL, active neoplastic processes or paraneoplastic syndromes, elevated concentrations of inflammatory markers—C-reactive protein concentration >5 mg/L or total white blood count exceeding 10 × 10^3^ cells/µL, chronic kidney disease (stages III–V), calcium and phosphorus metabolism disorders as well as vitamin D ingesting as a diet supplement or a medication.

### 2.2. Comorbidities Assessment

The patients enrolled in this study were measured in weight and height to diagnose obesity or overweight according to WHO criteria [43]. Based on the 2021 European Society of Hypertension practice guidelines for office and out-of-office blood pressure measurement, hypertension was defined as blood pressure greater than 140/90 mmHg in-office measurement [44]. Blood samples for laboratory tests were drawn through cephalic vein access and then examined in the hospital laboratory using standard clinical-chemical analysis. Type 2 diabetes mellitus was diagnosed based on the 2019 ESC Guidelines on diabetes, pre-diabetes, and cardiovascular diseases criteria [45], defining t2DM as having fasting blood glucose levels exceeding ≥ 7.0 mmol/L (126 mg/dL) or having symptoms of hyperglycemia (e.g., frequent urination, increased thirst, fatigue, acetone breath, nausea) accompanied with random blood glucose levels ≥ 200 mg/dL (≥11.1 mmol/L) or blood glucose at 120 min during an oral glucose tolerance test (OGTT) ≥ 200 mg/dL (≥11.1 mmol/L). Patients were diagnosed with hyperlipidemia if their lipid profile did not meet treatment targets for their respective risk level according to the 2019 ESC/EAS Guidelines for the management of dyslipidaemias [46].

Vitamin D serum concentrations were measured with the DiaSorin LIAISON^®^ 25 OH Vitamin D TOTAL Assay (Stillwater, MN, USA). It utilizes chemiluminescent immunoassay method, achieving a detection range between 4 and 150 ng/mL, with a precision equal to 5.0% of the coefficient of variation (CV), and a standard deviation of accuracy equaling 1.2% [47]. This test has a very good agreement strength (as defined by Cohen’s kappa coefficient) with Elecsys Vitamin D Total Assay, a test previously approved for clinical use for the Endocrine Society’s reference values for vitamin D deficiency (mentioned below) [48,49,50]. The vitamin D concentrations measured by the LIAISON^®^ 25 OH Vitamin D TOTAL Assay were measured in ng/mL, where 1 ng/mL equals 2.5 nmol/L [51].

The results of the vitamin D concentration were classified according to the Endocrine Society’s guidelines of clinical practice for vitamin D deficiency: concentrations <10 ng/mL, between ≥10 and <20 ng/mL, between ≥20 and <30 ng/mL, and ≥30 mg/mL were labeled severe deficiency, moderate deficiency, mild deficiency, and optimal concentration, respectively [25]. As patients were examined in different months of the year, a seasonal concentration deviation was observed (lower concentrations from November to April versus higher concentrations from May to October) [52,53]. This dependence is due to changes in the availability of the UVB from the sunlight in Warsaw, Poland (52°13′ N, 21°02′ E). The UVB-reliant synthesis of vitamin D in the human skin can occur efficiently only from May to October at this latitude [53].

Total blood count was measured in blood samples collected from the cephalic vein in di/tripotassium EDTA tubes by an automatic blood counter from this brand within two hours after the venepuncture. The NLR factor was defined as the quotient of absolute neutrophil count to absolute lymphocyte count.

### 2.3. Coronary Angiography

Coronary angiography, which uses X-rays and iodinated contrast to visualize coronary arteries, is the default method for stenosis assessment in CAD [54]. In this study, the examination was performed through access to the radial or femoral artery. The severity of CAD was classified with the Coronary Artery Surgery Study Score (CASSS) by three independent cardiologists; uncertain cases of moderate versus severe stenosis were resolved using fractional flow reserve (FFR). CASSS is a scale reflecting the stenosis of the arteries. A major coronary artery (right coronary artery, circumflex branch, or anterior descending branch) stenosis exceeding 70% resulted in assignment of one point. Left main coronary artery stenosis over 50% was rated as two points. CASSS is the sum of the points (0–3) reflecting CAD of between one and three vessels. ACS was diagnosed using criteria from the European Society of Cardiology guidelines, which is the increased concentration of markers of myocardial injury with the coexistence of at least one of the specified below: symptoms of stenocardia, changes in ECG suggesting ischemia, results of imaging tests depicting myocardial necrosis or coronary artery thrombus identification on coronary angiography [55].

### 2.4. Statistical Analysis

The data distribution were determined with a Shapiro–Wilk test. Comparison of continuous variables in the results was evaluated with a Mann–Whitney test or a *t*-test. Differences between prevalence in selected groups were determined with a Pearson’s chi-square test or a Fisher’s exact test. The NLR was compared between patients with different CASS scores using Kruskal–Wallis analysis. The relationship between the selected variables was analyzed with a Spearman’s correlation coefficient (R). Statistical significance was recognized if a two-sided *p*-value <0.05. Statistical analysis was completed with Statistica 13 (StatSoft Inc., Tulsa, OK, USA) and the figures were drawn with GraphPad Prism 8.0 (GraphPad Software, San Diego, CA, USA).

## 3. Results

### 3.1. Participants’ Characteristics

Details on the study group are presented in Table 1.

Table 2, published also in [37], presents a comparison between patients with stable CAD and ACS in measured parameters. ACS and stable CAD were the cause of hospitalization in 60% and 40% of patients, respectively. Significant differences between patients with stable CAD and patients with ACS were observed for vitamin D level and LDL level. Statistically significant disproportions between the subgroups of patients were noticed in terms of sex, diabetes, hyperlipidemia, and smoking. Details on factors influencing the cause of hospitalization were presented elsewhere [37].

### 3.2. Difference in NLR between Patients with Stable CAD and Patients with ACS

**Figure 1:**
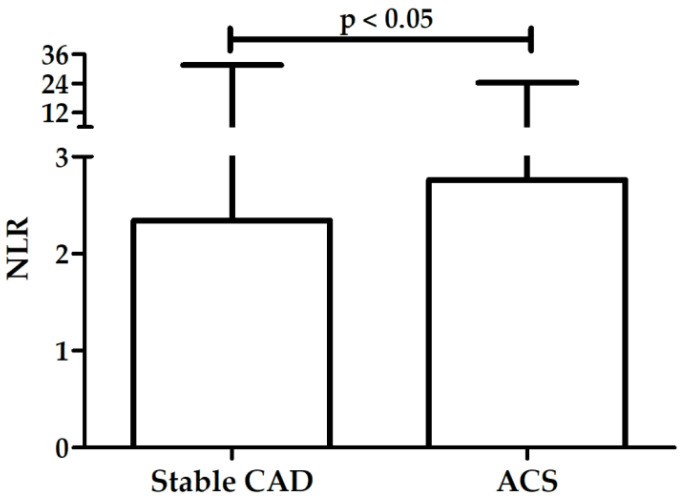
Difference in NLR between patients with stable CAD and patients with ACS, box—median, whiskers—range.

There was a significant difference in NLR between patients with stable CAD and patients with ACS (median: 2.3, range: 0.03–31.6 vs. median: 2.8, range: 0.96–24.3; *p* < 0.05; Figure 1).

### 3.3. Association between NLR and Severity of CAD

**Figure 2:**
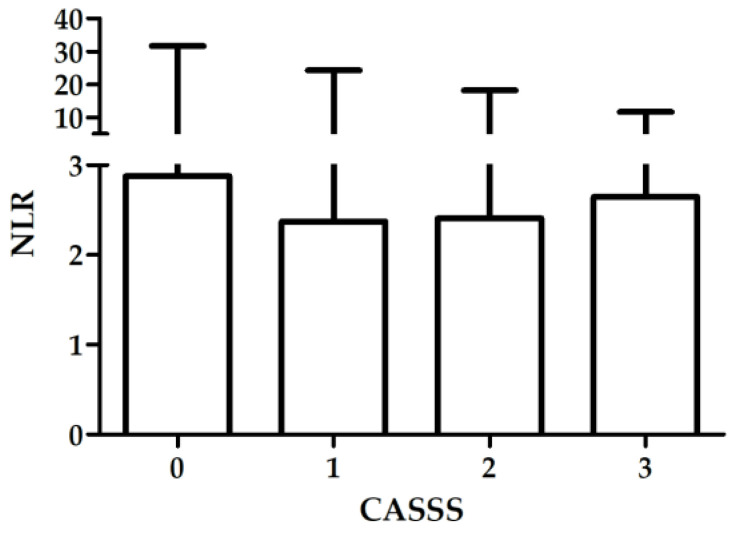
Association between NLR and severity of CAD in the whole group, box—median, whiskers—range.

There was no association between NLR and severity of CAD in the whole group (H = 5.46; *p* = 0.14; Figure 2), nor in the patients with stable CAD (H = 2.93; *p* = 0.40) or ACS (H = 3.33; *p* = 0.34). Results are presented in Table 3.

### 3.4. Correlation between Vitamin D Levels and NLR

**Figure 3:**
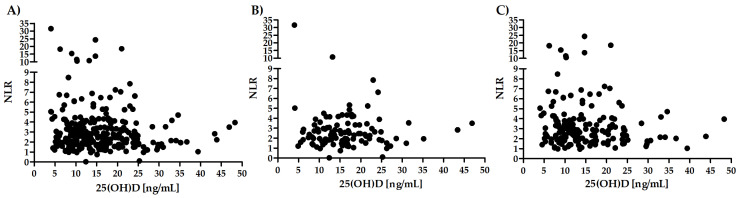
Correlation between the 25(OH)D level and NLR in the whole group (**A**) and subgroups of patients with stable CAD (**B**) and ACS (**C**).

There was a lack of a significant correlation between the level of 25(OH)D and NLR in the whole group (R = −0.07; *p* = 0.29; Figure 3A) as well as in the subgroups of patients with stable CAD (R = 0.02; *p* = 0.84; Figure 3B) and ACS (R = −0.08; *p* = 0.30; Figure 3C).

## 4. Discussion

This study analyzed NLR and serum vitamin D concentration in a group of patients with a history of MI as part of a research project aiming to describe the relationship between vitamin D level and CAD severity. In previous studies, we showed significantly decreased serum vitamin D concentration in patients hospitalized for ACS compared to those diagnosed with stable CAD [38] and no significant differences in platelet activity between these two groups of patients [37]. Vitamin D concentration did not correlate with MPV and P-LCR [37]. Results presented in this study suggest that patients with consecutive MI have significantly elevated NLR compared to a group of patients diagnosed with stable CAD. There were no significant differences in NLR between groups of patients with different stages of CAD (CASSS 0–3), however, it should be noted that nominally the highest NLR was observed in patients with ACS and three-vessel CAD (See Table 3). Similarly, for selected platelet activity parameters [37], there was no significant correlation between vitamin D level and NLR in the analyzed study group. 

A strong correlation was previously described between hematologic parameters and the risk of adverse cardiovascular events as an effect of inflammation and hypoxemia [56,57]. Leukocytes, especially lymphocytes, play an important role in modulating the inflammation process, resulting in lymphopenia that aggravates the atherosclerotic process [58]. However, a high neutrophil count can lead to increased blood coagulability, influencing platelets and vascular endothelial cells and resulting in damage to microcirculation vessels [59,60].

NLR is an affordable and accessible marker of inflammation [61]. Due to being a ratio of two different, but complementary immunological pathways, its insensitivity to physiological states (dehydration, exertion) is an additional advantage in comparison to other cardiovascular risk markers, e.g., white blood or neutrophil count [20]. NLR was described as an independent outcome predictor in stable CAD, as there is a relationship between NLR and the diagnosis and staging of stable CAD [19,62]. A threshold value of 2.54 for NLR with sensitivity and specificity of 74% and 53%, respectively, was suggested as a prognostic marker of severe atherosclerosis before coronary artery angiography [63]. The meta-analysis of 17 studies, including more than 7000 cases, claimed that NLR is a useful biomarker of the severity of CAD and the presence of significant coronary stenosis [64]. Similar results were reported by Maleki et al., who found a correlation between NLR and the severity of CAD measured by the SYNTAX scale in patients with a history of non-ST segment elevation myocardial infarction (NSTEMI) [65]. However, they claimed that the TIMI risk score had an advantage over NLR in the prediction of the SYNTAX score. As our results indicate the lack of a correlation between NLR and CAD severity seems to contradict those mentioned above, we think it may be caused by specific characteristics of our cohort of patients, as nearly 95% of them had significant stenosis of at least one main coronary artery in addition to a history of MI. Although our results suggest that there are no significant differences in NLR levels in patients with different CASSS, it is worth acknowledging that the highest NLR was presented by patients with a consecutive ACS and three-vessel CAD (CASSS 3) (See Table 3).

The relationship between NLR and a consecutive ACS found in our cohort was also observed by other authors. In an observational study, NLR was suggested as a diagnostic tool for patients with thoracic pain [66] and NLR > 7.4 was correlated with a greater than twofold increase in the risk of ACS in patients with chronic kidney disease and elevated markers of myocardial necrosis [67]. In non-ST segment elevation ACS, NLR was directly associated with the SYNTAX score and could be used to estimate the risk of complications. In a group of patients with a history of ACS, NLR was a strong predictor of short- and long-term mortality. In NSTEMI, NLR > 4.7 was associated with a nearly threefold increase in mortality risk compared to patients with NLR < 3 [20]. In patients with ST-elevation myocardial infarction, NLR > 3.7 was a prognostic marker of a serious complication—ventricular free wall rupture [68]. After PCI, NLR was considered an independent indicator of coronary flow since it was positively correlated with corrected TIMI frame count [69]. Other studies suggested NLR as a marker of a higher risk of ventricular arrhythmia (NLR 3.79 vs. 1.56) [70] and long-term mortality, independent of indications for PCI [71,72]. In patients with a history of coronary artery bypass graft, elevated NLR in the preoperative and postoperative period (median 3.0 and 9.2, respectively) was a predictor of atrial fibrillation [73] and correlated well with the risk of mortality or transplantation in patients with severe congestive heart disease [74].

The availability of data on the dependence between NLR and serum vitamin D concentration is very limited. Akbas et al. demonstrated the inverse correlation of serum vitamin D concentration and other markers of inflammation, including NLR [75]. Verdoia et al. drew similar conclusions, indicating a significant association between vitamin D concentration and NLR in patients with and without diabetes mellitus [35]. These studies suggest that vitamin D influences NLR as a marker of subclinical inflammation resulting in the progress of atherosclerosis [35,75], which is not supported by our results. Considering the documented role of vitamin D as a hormone regulating the expression of a multitude of genes [76], the association with cardiovascular disease and risk factors as well as the antioxidant and anti-inflammatory, thus anti-atherosclerotic properties of calcitriol [77], we suggest further research on this subject. The role of vitamin D in the pathogenesis of stable CAD and its complications was suggested in our previous study, as significantly lower concentrations of vitamin D were found in patients with diagnosed ACS [2]. This was also proven in cohort studies, since vitamin D concentrations under 15 ng/mL were associated with a twofold increase in the risk of ACS and levels above 7.3 ng/mL were associated with a reduction in major adverse cardiovascular events (excluding death) in nearly half of the patients [78].

There are some limitations to this study. First, it was carried out on a relatively small group of patients lacking demographic diversity, as the majority of patients resided in a city. Second, the observational and cross-sectional character of this study inherently limited the scope of causation analysis of the variables. Furthermore, the CAD severity classification based on angiography cannot take into account the stabilizing effect of coronary artery calcification. Moreover, the influence of comorbidities, hypolypemizing drugs, smoking, and others with an ability to influence NLR significantly, were not taken into account. Apart from the traditional markers of inflammation (CRP, WBC), the study did not include other commonly used indicators such as interleukin 6 or ferritin.

Chronic inflammation, including a cellular immune response, plays a key role in the pathogenesis of atherosclerosis and its complications [79]. Recent studies carried out on data of patients with arthritis or pneumonia suggest that NLR could provide better insight into subclinical inflammation compared to commonly used tests (C-reactive protein, white blood cell count) [80,81,82,83,84]. However, the role of NLR as an indicator of inflammation in the coronary artery wall has not yet been established and requires further research.

We suggest considering carrying out well-designed studies to determine the role of the NLR indicator in predicting another infarction in patients with CAD diagnosis and a history of MI. The relevance of serum vitamin D concentration in patients with consecutive MI, as well as its influence on immune response and inflammatory markers also needs further well-designed research.

## 5. Conclusions

In a group of patients with recurrent MI there is an elevation of NLR. Although no correlation was found between serum vitamin D concentration and NLR, the lowest vitamin D concentrations and the highest NLR were observed in patients with ACS. The role of vitamin D and NLR in CAD pathogenesis and its complications needs further research.

## Figures and Tables

**Table 1:** Participants’ characteristics.

Variable	Values
Age [years]	67.0 ± 11.2
BMI [kg/m^2^]	28.0 ± 4.4
Diabetes mellitus [yes/pre-diabetes/no]	100/10/158
Hyperlipidemia [yes/no]	145/107
Hypertension [yes/no]	233/35
Smoking [active/former smoker/no]	85/28/155
Season during the examination [November to April/May to October]	200/68
CASSS [0/1/2/3]	13/79/83/93
Serum 25(OH)D [ng/mL]	14.0 (4.0–48.3)
Neutrophils [thousand cells/µL]	4.9 (1.4–23.8)
Lymphocytes [thousand cells/µL]	1.9 (0.3–189.0)
NLR	2.6 (0.03–31.6)

BMI—body mass index; CASSS—Coronary Artery Surgery Study Score; NLR—Neutrophil-to-lymphocyte ratio.

**Table 2:** Comparison of the obtained parameters between patients with stable coronary artery disease and patients with acute coronary syndrome.

Variable	Stable CAD	ACS	*p*-Value
Number of participants	108	160	-
Sex (♀/♂)	27/81	60/100	<0.05
Age (years)	68.4 ± 9.4	66.1 ± 12.2	0.10
BMI (kg/m^2^)	27.7 ± 4.3	28.3 ± 4.6	0.34
BMI class (1/2/3) *	28/52/28	33/59/47	0.41
Diabetes (No/Yes/prediabetes)	63/36/9	95/64/1	<0.01
TC (mg/dL)	162.5 (84.8–327.3)	171.9 (70.9–338.3)	0.07
HDL (mg/dL)	46.5 (14.6–113.2)	44.5 (19.5–92.9)	0.11
LDL (mg/dL)	81.9 (27.3–257.9)	101.4 (24.4–244.3)	<0.05
TG (mg/dL)	111.8 (37.9–417.0)	115.4 (42.6–391.8)	0.58
Hyperlipidemia (No/Yes)	54/50	53/95	<0.05
Hypertension (No/Yes)	15/93	20/140	0.74
Smoking (No/Yes/Ex-smokers)	62/24/22	93/61/6	<0.001
CASSS (0/1/2/3)	6/25/39/38	7/54/44/55	0.24
Serum 25(OH)D (ng/mL)	15.8 (4.0–46.9)	13.1 (4.0–48.3)	<0.05
Season of the examination (November to April/May to October)	78/30	122/38	0.46

*—1—<25. 2—25–30. 3—> 30.

**Table 3:** NLR in patients with different stages of CAD.

NLR	CASSS			
	0	1	2	3
All patients	2.9 (1.1–31.6)	2.4 (1.0–24.3)	2.4 (0.03–18.2)	2.7 (1.0–11.6)
Stable CAD	2.5 (1.1–31.6)	2.4 (1.0–7.9)	2.0 (0.03–5.3)	2.5 (1.2–10.8)
ACS	2.9 (2.2–18.6)	2.4 (1.0–24.3)	2.6 (1.1–18.2)	3.1 (1.0–11.6)

CASSS—Coronary Artery Surgery Study Score; NLR—Neutrophil-to-lymphocyte ratio.

## Data Availability

Data can be provided by the corresponding author upon reasonable request.
